# Using quantile regression to investigate racial disparities in medication non-adherence

**DOI:** 10.1186/1471-2288-11-88

**Published:** 2011-06-06

**Authors:** Mulugeta Gebregziabher, Cheryl P Lynch, Martina Mueller, Gregory E Gilbert, Carrae Echols, Yumin Zhao, Leonard E Egede

**Affiliations:** 1 Center for Disease Prevention and Health Interventions for Diverse Populations, Ralph H. Johnson Veterans Affairs Medical Center, Charleston, USA; 2 Center for Health Disparities Research, Department of Medicine, Medical University of South Carolina, Charleston, USA; 3 Division of Biostatistics & Epidemiology, Medical University of South Carolina, Charleston, USA; 4 College of Nursing, Medical University of South Carolina, Charleston, USA

**Keywords:** Medication adherence, Quantile regression, Diabetes, Health disparities

## Abstract

**Background:**

Many studies have investigated racial/ethnic disparities in medication non-adherence in patients with type 2 diabetes using common measures such as medication possession ratio (MPR) or gaps between refills. All these measures including MPR are quasi-continuous and bounded and their distribution is usually skewed. Analysis of such measures using traditional regression methods that model mean changes in the dependent variable may fail to provide a full picture about differential patterns in non-adherence between groups.

**Methods:**

A retrospective cohort of 11,272 veterans with type 2 diabetes was assembled from Veterans Administration datasets from April 1996 to May 2006. The main outcome measure was MPR with quantile cutoffs Q1-Q4 taking values of 0.4, 0.6, 0.8 and 0.9. Quantile-regression (QReg) was used to model the association between MPR and race/ethnicity after adjusting for covariates. Comparison was made with commonly used ordinary-least-squares (OLS) and generalized linear mixed models (GLMM).

**Results:**

Quantile-regression showed that Non-Hispanic-Black (NHB) had statistically significantly lower MPR compared to Non-Hispanic-White (NHW) holding all other variables constant across all quantiles with estimates and p-values given as -3.4% (p = 0.11), -5.4% (p = 0.01), -3.1% (p = 0.001), and -2.00% (p = 0.001) for Q1 to Q4, respectively. Other racial/ethnic groups had lower adherence than NHW only in the lowest quantile (Q1) of about -6.3% (p = 0.003). In contrast, OLS and GLMM only showed differences in mean MPR between NHB and NHW while the mean MPR difference between other racial groups and NHW was not significant.

**Conclusion:**

Quantile regression is recommended for analysis of data that are heterogeneous such that the tails and the central location of the conditional distributions vary differently with the covariates. QReg provides a comprehensive view of the relationships between independent and dependent variables (i.e. not just centrally but also in the tails of the conditional distribution of the dependent variable). Indeed, without performing QReg at different quantiles, an investigator would have no way of assessing whether a difference in these relationships might exist.

## Background

Diabetes is a chronic debilitating illness that affects approximately 24 million people in the United States [1]. Medication adherence is an important component of good diabetes care and medication non-adherence is associated with poor glycemic control [2,3], increased health utilization [4,5], increased health care costs [6,7], and increased risk of death [5]. African Americans and other ethnic minority groups have higher prevalence of diabetes and are at increased risk for poor outcomes from diabetes [1]. Multiple recent studies have shown that ethnic minority groups with diabetes have poorer glycemic, lipid, and blood pressure control compared to Whites [8]. There are also data that suggest a correlation between ethnic differences in diabetes outcomes (e.g., glycemic, lipid, and blood pressure control) and ethnic differences in medication adherence [9]. Therefore, medication non-adherence is an important risk factor for poor diabetes outcomes, especially in ethnic minority groups.

Several methods exist to assess medication adherence including patient self-report, pill counts, physician/nurse report, pharmacy refill data, electronic monitoring, and biological assays [10]. The most commonly used methods use pharmacy refill data and provide reliable estimates of medication adherence [10]. Common methods for assessing medication non-adherence with pharmacy refill data include continuous measure of medication acquisition (CMA), continuous multiple intervals of oversupply (CMOS), medication possession ratio (MPR), and medication refill adherence (MRA), which have all been shown to be identical in terms of measuring adherence to prescription refills over a study period [11].

While the literature on ethnic/racial disparities on medication adherence is scant, some studies using pharmacy refill data from administrative databases have documented ethnic differences in medication adherence among individuals with diabetes [12-14]. However, the magnitude of these racial/ethnic differences is unclear, especially across ranges of medication adherence (e.g. 40% vs. 60% vs. 80%). In addition, it is not clear if the findings of prior studies are reliable given some methodological weaknesses. For example, most prior studies used traditional regression methods that may not be valid if certain assumptions are not satisfied. Some studies used linear regression, which requires the residuals to be normally distributed and homoscedastic [5,9]. Others have used logistic regression after categorization of the outcome [4,12,14], which could lead to arbitrary choice of categories such that results could be sensitive to choice of cutoff values. These methods also may not capture the effect of covariates on the entire distribution of the response variable.

While both linear and logistic regression focus on differences in means associated with covariates, quantile regression allows for studying different directions of the effects of a covariate on different parts of the distribution (lower and upper tails, middle part). Furthermore, quantile regression makes use of the full information of data in contrast to logistic regression, which is usually associated with a loss of information due to transformation of the response MPR into a categorical variable (e.g., binary variable with cutoff at 80%). More importantly, MPR is a quasi-continuous variable that takes on values that are bounded (i.e., have lower and/or upper bounds) and hence traditional methods that use mean changes of the dependent variable with changes in the independent variables may fail to discern differential patterns in non-adherence across racial/ethnic groups. Therefore, the aims of this study were twofold. First, was to examine racial differences in medication non-adherence using quantile regression. Second, was to demonstrate through empirical evidence how choice of a regression method (e.g., QReg, OLS or GLMM) could result in different conclusions for response variables like MPR, which usually have skewed distributions and take on bounded values. We hypothesized that QReg provides estimates of the effect of covariates on the conditional quantiles of MPR, leading to a more complete picture of the differences between race/ethnicity groups over the entire distribution of MPR including the tails and center of the conditional distribution.

## Methods

We created a cohort of veterans with type 2 diabetes from a Veterans Administration (VA) facility in the Southeastern United States using multiple patient and administrative files from the Veterans Health Administration (VHA) Decision Support System (DSS) files linked by Social Security Number (SSN). The study period was from April 1996 to May 2006 with an average follow up period of 5.4 years. The datasets were merged, cleaned and then used as the final dataset for analysis. Veterans with type 2 diabetes were identified based on having at least two ICD-9 codes for diabetes (250.xx) in either outpatient or inpatient files and having two or more visits each year since diagnosis based on a previously validated algorithm [15]. The datasets were merged to create a subset that only included individuals with complete adherence data, resulting in a cohort of 11,272 veterans with type 2 diabetes, of which 5,307 were non-Hispanic White (NHW), 3,061 were non-Hispanic Black (NHB), 51 were Hispanic and 1,879 were identified as Other ethnic/racial group. There were also 974 (8.6%) with missing or unknown race/ethnicity information. The study was approved by our institutional review board (IRB) and local VA Research and Development committee.

### Outcome Measures

The primary outcome was the mean medication possession ratio (MPR). MPR informs patient medication adherence by providing the ratio of the number of days of medication supplied within a refill interval to the number of days in a specified refill interval [16,17]. We calculated the number of eligible days per medication within each 90-day refill period per patient. We considered supply of insulin and oral hypoglycemic agents (VA classes HS501 and HS502, respectively). The sum of eligible days served as the denominator for the MPR calculation [18]. The average MPR was calculated over the follow up period from 1996-2006. Prescriptions that became inactive during that time period did not contribute to the MPR calculation. We chose 90-day intervals because veterans typically have a 90-day of supply of medications mailed to their homes. If the MPR exceeded 100%, it was set to 100%.

### Primary Covariate

The primary covariate of interest was race/ethnicity classified as NHW, NHB, and Other (including unknown and missing).

### Demographic Variables

We controlled for three demographic variables in addition to the primary covariate. Age at baseline was treated as a continuous variable and centered at its mean value. Marital status was classified as never married, married (reference category), or separated/widowed/divorced. Employment was classified as employed, not employed (reference category), or retired.

### Medical Comorbidity

Cancer, congestive heart failure (CHF), coronary heart disease (CHD), hypertension, and stroke were defined based on enhanced ICD-9 codes using validated algorithms [19] and coded as 0 or 1 based on presence or absence of history of the disease at baseline.

### Psychiatric Comorbidity

Six psychiatric comorbidities including bipolar disorder, generalized anxiety disorder, major depressive disorder, post-traumatic stress disorder, psychotic disorders, and substance use disorder were defined as present (1) or absent (0) at baseline based on enhanced ICD-9 codes using validated algorithms [19].

### Statistical analysis

First, we examined the characteristics of the sample through univariate analysis. This step was followed by pre-model building analysis, which included testing whether each covariate was individually associated with the outcome. To assess whether the relationship between age and MPR was non-linear, we examined the significance of a quadratic term for age. Next, a final model investigating the association between MPR and race/ethnicity was developed adjusting for all covariates such as demographics, medical comorbidities, and psychiatric comorbidities.

For quantile regression analysis, the response variable, MPR, was defined as the quantile of the mean medication possession ratio for each individual averaged over the study period. The specifications of the unconditional quantiles were made in two different ways: Scenario 1) the quantiles were specified based on clinically meaningful specific MPR cutoff values: Q1 = 0.40, Q2 = 0.60, Q3 = 0.80, Q4 = 0.90 where the values corresponded to the 2^nd^, 4^th^, 15^th ^and 27^th ^percentiles of the distribution of MPR and Scenario 2) the quantiles were based on the distribution of MPR values where the 5^th^, 10^th^, 15^th^, 25^th ^and 50^th ^percentiles were considered. These unconditional percentiles corresponded to MPR cutoff values of Q1 = 0.66, Q2 = 0.75, Q3 = 0.80, and Q4 = 0.88 and Q5 = 0.97, respectively.

Quantile regression is used to model the effects of covariates on the conditional quantiles of a response variable [20]. This approach is a robust method that makes no distributional assumption about the error term in a model. It is also robust to extreme points in the response space (outliers) but not to extreme points in the covariate space (leverage points). Confidence intervals for the estimated parameters in QReg are based on inversion of a rank test [21,22].

#### Quantile Regression Model

For a random response variable Y with probability distribution function F(y) = Prob (Y ≤ y), the τ^th ^quantile of Y is defined as the inverse function Q(τ) = inf {y : F(y) ≥ τ} where 0 <τ < 1. Let X = (x_1_, ..., x_n_) denote the matrix consisting of n observed vectors of the random vector X, and let Y = (y_1_, ..., y_n_) denote the n observed responses. The model for linear quantile regression is given by *y_i _*= *x_i_β_τ _*+ *ε_i_*, where *β_τ _*= (*β*_1*τ*_, ..., *β_pτ_*) is the unknown p-dimensional vector of parameters and *ε *= (*ε*_1_,..., *ε*_n_) is the n dimensional vector of unknown errors (Assumption: the *τ*th quantile of *ε_i _*is zero). The *β_τ _*is a solution of,

The special case τ = 0.5 is equivalent to median regression. We used the finite smoothing algorithm [23,24] to compute the solution of this equation so that the Newton-Raphson algorithm could be used iteratively to obtain the solution after a finite number of loops. The regression coefficient at a given quantile (*β_τ_*) indicates the effect on Y of a unit change in X, assuming that the other factors are fixed.

Both unadjusted and covariate adjusted models were fitted with MPR as the response variable and race/ethnicity as primary variable of interest. Since our sample size is sufficiently large, the final model was adjusted for all covariates including demographic variables such as age, gender, marital status, employment status and medical and psychiatric comorbidities [25]. All models were assessed for goodness-of-fit using residual analysis. In addition, QReg was assessed using robust multivariate location and scale estimates for leverage point detection [26].

PROC QUANTREG in SAS 9.2 (SAS Institute Inc., Cary NC) was used to compute the regression models and to conduct statistical inferences on the estimated parameters. Verification for all QReg models was performed using the R [27] quantreg package.

#### Ordinary Least Squares (OLS)

SAS Proc GLM was used to estimate the parameters of a multiple regression model where the errors for different observations were assumed to be uncorrelated with identical variances (homoscedastic). Under these assumptions, OLS provides estimates of the linear parameters that are unbiased and have minimum variance among linear estimators. Residual plots were used to assess these assumptions but they did not hold true for our data.

#### Generalized linear Mixed Model (GLMM)

This model extends the above model by allowing a more flexible specification of the covariance matrix of the error terms. In other words, it allows for both correlation and heterogeneous variances, although requires normality assumption [28] which did not hold true for our data. SAS Proc GLIMMIX was used to estimate the parameters of a linear mixed model with a random intercept. This specification allowed different subjects to have different baseline MPR values. The same sets of covariates were used in OLS, GLMM and QReg.

### Comparison of statistical methods (QReg, OLS, GLMM)

The second aim was addressed using empirical studies based on re-sampling of the data with replacement. Traditionally, Monte-Carlo simulation studies based on data generated from statistical models have been used for this kind of comparative study. Resampling has the advantage that the data in resampled datasets are based on observations from real patients [29] and thus reflect the appropriate level of diversity and variability found in realistic populations [30,31]. Sampling with replacement was used since our dataset can be considered large to permit numerous samples of reasonable size to obtain stable conclusions within the smaller samples. Each dataset in the resampling study consisted of 5,000 patients, which represents many of the typical studies that use regional VA data. In order to robustly and accurately estimate the parameters, a total of 10,000 bootstrap replications were performed. The final estimates of the parameters and their standard errors were obtained using means and standard deviations of the 10,000 parameter estimates. Additionally, we computed exact percentiles (e.g., 97.5%; 2.5%) for constructing empirical confidence intervals.

## Results

**Figure 1:**
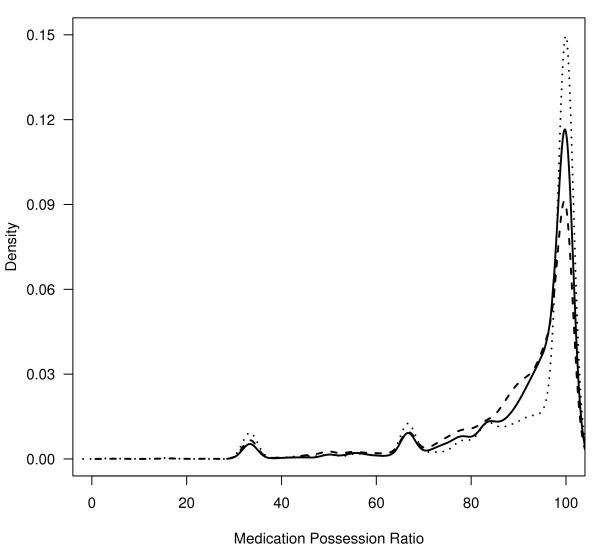
**Distribution of Medication Possession Ratio (MPR) by Race/Ethnicity (Non-Hispanic White, Non-Hispanic Black, Other groups)**. dotted line = Other, dashed line = Non-Hispanic Black, solid line = Non-Hispanic White

Table 1 shows the socio-demographic characteristics for the 11,272 veterans with type 2 diabetes included in this sample. Approximately 97% were male with 47% being NHW and 27% NHB. The mean age was 66 years. The most prevalent medical comorbidities were hypertension (26%), CHD (14%) and CHF (8%). The most prevalent psychiatric comorbidities were substance use disorder (14%) and MDD (8%). During the study period the overall mortality was 16%. The mean HbA1c value was 7.0% (sd = 0.9%). Most Veterans (88.4%) had HbA1c values ≤ 8.0%. The mean (sd) MPR values for NHW, NHB and Others were 91.2% (0.2), 88.7% (0.3) and 90.7% (0.3), respectively. Figure 1, a density plot of MPR by race, shows the highly skewed nature of the distribution of MPR by race/ethnicity.

**Table 1:** Sample Characteristics by Race and Ethnicity (n = 11,272)

	Race/Ethnicity Category			
**Variable**	**All**	**NHW**	**NHB**	**Other**

Age (years mean, sd)	66 (11.6)	68 (10.7)	64 (12.3)	66 (11.7)

Male	97.3	97.8	96.6	97.2

Female	2.7	2.2	3.4	2.8

Married	65.2	67.2	58.3	68.8

Divorced	28.6	28.1	31.6	26.3

Never Married	6.2	4.7	10.1	4.8

Unemployed	48.2	48.8	53.0	42.0

Retired	30.8	32.7	25.4	33.1

Employed	20.8	18.4	21.6	24.5

Cancer	5.0	5.2	7.5	2.1

CHD	13.9	20.0	12.4	4.6

CHF	8.0	10.0	9.3	3.1

Hypertension	25.7	29.2	33.8	10.7

Stroke	3.0	4.1	3.1	1.0

HbA1c 8+	11.6	10.0	15.5	10.3

HbA1c (% mean, sd)	7 (0.9)	7 (0.9)	7 (1)	7 (0.9)

Bipolar Disorder	1.9	2.2	2.5	0.6

Generalized Anxiety Disorder	2.2	3.1	1.9	0.7

Major Depressive Disorder	7.8	8.8	10.5	3.0

Post Traumatic Stress Disorder	5.1	4.6	7.9	3.0

Psychotic Disorder	2.4	1.7	4.9	1.1

Substance Use Disorder	14.4	14.7	21.4	6.4

Dead	16.4	19.0	16.5	11.7

MPR (mean, sd)	90.4 (0.2)	91.2 (0.2)	88.7 (0.3)	90.7 (0.3)

MPR (median, IQR)	97.1 (13)	97.1 (11)	95.0 (16)	100 (11.9)

NHW-non-Hispanic white; NHB-non-Hispanic black;

CHD-coronary heart disease; CHF-congestive heart failure;

HbA1c-glycosolated hemoglobin (%);

MPR-medication possession ratio

MPR values are reported in percentages.

**Figure 2:**
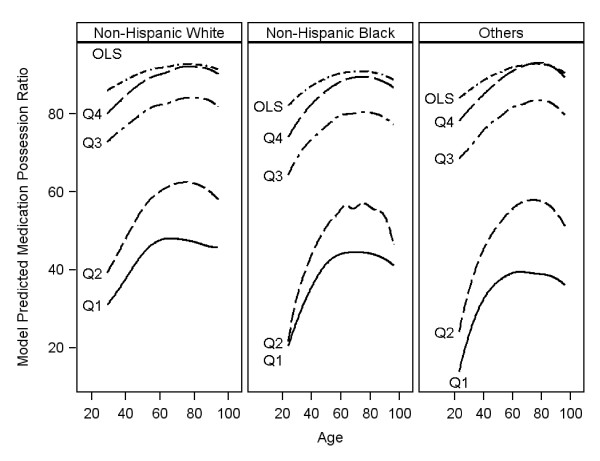
**Distribution of Predicted Mean Medication Possession Ratio (MPR) by age for each type of model (Quantile Regression versus OLS)**. OLS = ordinary least squares. Q_i _= ith quantile (i = 1,.4): Quantiles are based on unconditional MPR cutoff values: 0.4, 0.6, 0.8 and 0.9.

We focus the description of quantile regression results on Scenario 1 since the results on Scenario 2 were qualitatively similar and also because most clinicians are interested in this scenario. In Figure 2, results comparing quantile regression with ordinary least square (OLS) regression are shown. While the curves across age for OLS are similar for all three race groups showing smaller racial/ethnic differences in mean MPR that decreased with age, the curves for QReg clearly indicate differences in MPR across race groups particularly in the lower quantiles of the MPR distribution. The differences are more pronounced in the three lower quantiles. The difference in MPR disappears with higher age in almost all the quantiles of medication adherence.

In Table 2, the intercept in the first panel is interpreted as the estimated conditional quantile function of the MPR distribution of a type 2 diabetes patient who was female, NHW, married, unemployed, with no history of medical or psychiatric comorbidity and had an average age of the study population (age = 66 years, since age was centered at 66). In this adjusted QReg model, NHW had consistently higher MPR over all quantiles compared to NHB, and over quantiles 1 and 2 compared to Other (other racial groups). Compared to NHB, NHW had 3.4% (p < 0.11) higher MPR in the first quantile (Q1), 5.4% (p < 0.01) in the second quantile (Q2), 3.1% (p < 0.001) in the third quantile and 2.0% (p < 0.001) in the fourth quantile (Q4). Similarly, compared to Other race groups, NHW had 6.3% (p < 0.001) higher MPR in the first quantile (Q1) and 3.8% (p = 0.09) in the second quantile (Q2). The mean MPR values were also higher for NHW compared to NHB (1.4%, p < 0.001) as shown in the results for OLS and GLMM. However, the mean MPR difference between NHW and Other races was not significant (0.10%, p = 0.74).

**Table 2:** Adjusted parameter estimates (β) and p-values for quantile regression, ordinary least-squares regression, and the generalized linear mixed model

	*QReg*								*OLS*		*GLMM*	
				
	***Quantile 1***		***Quantile 2***		***Quantile 3***		***Quantile 4***					
***Parameter***	*β*	*P*	*β*	*P*	*β*	*P*	*β*	*P*	*β*	*P*	*β*	*P*

**Intercept**	38.2	0.00	49.2	0.00	72.1	0.00	82.6	0.00	88.2	0.00	89.5	0.00

**NHB**	-3.42	0.11	-5.42	0.01	-3.1	0.00	-2.0	0.00	-1.4	0.00	-1.3	0.00

**Other**	-6.33	0.00	-3.75	0.09	-0.9	0.34	0.5	0.36	-0.1	0.74	-0.1	0.70

**NHW (REF)**												

**Male**	-11.4	0.04	-7.00	0.22	0.4	0.86	1.1	0.42	-0.5	0.58	-0.4	0.62

**Age**	0.24	0.01	0.27	0.00	0.2	0.00	0.1	0.00	0.1	0.00	0.1	0.00

**Age-squared**	-0.01	0.14	-0.01	0.04	0.0	0.11	0.0	0.00	0.0	0.00	0.0	0.00

**Never Married**	-2.84	0.45	-7.62	0.05	-0.5	0.79	-1.8	0.06	-0.9	0.16	0.9	0.13

**Divorced**	-3.37	0.09	-5.35	0.01	-2.9	0.00	-2.6	0.00	-1.4	0.00	1.2	0.00

**Married (REF)**												

**Employed**	5.49	0.02	4.52	0.07	2.8	0.01	1.1	0.06	1.3	0.00	0.6	0.15

**Retired**	-1.03	0.61	2.04	0.34	1.9	0.04	1.3	0.01	0.6	0.06	-0.6	0.04

**Unemployed (REF)**												

**Cancer**	-8.24	0.05	-13.0	0.00	-3.1	0.10	-0.4	0.70	-1.4	0.05	-1.0	0.11

**CHD**	14.8	0.00	8.2	0.01	0.0	1.00	0.4	0.65	0.7	0.19	0.5	0.31

**CHF**	1.37	0.71	0.3	0.93	-1.0	0.54	-2.0	0.04	-0.9	0.16	-0.9	0.08

**Hypertension**	0.46	0.86	-0.5	0.85	-0.8	0.51	-1.6	0.02	-1.1	0.01	-0.9	0.02

**Poor HbA1c**	12.2	0.00	7.1	0.01	-0.6	0.58	-2.7	0.00	-1.0	0.02	-5.0	0.00

**Stroke**	-1.60	0.75	-4.7	0.37	-0.5	0.81	-0.7	0.58	-0.6	0.47	-0.5	0.47

**Bipolar**	-4.16	0.54	-5.1	0.47	2.3	0.45	-0.6	0.72	-0.2	0.84	0.1	0.93

**GAD**	1.53	0.79	-1.1	0.86	3.7	0.16	1.6	0.28	0.9	0.36	0.6	0.45

**Psychoses**	7.28	0.23	6.8	0.28	-3.4	0.21	0.9	0.57	0.0	1.00	-0.5	0.53

**PTSD**	-3.30	0.40	-1.6	0.70	2.6	0.14	1.4	0.18	0.7	0.30	0.9	0.12

**Substance Use**	7.00	0.01	5.78	0.03	2.6	0.02	1.5	0.02	1.0	0.02	0.8	0.03

NHB = Non-Hispanic Black

NHW = Non-Hispanic White (Reference)

Other = Other race groups

β = Parameter Estimate

P = p-value

OLS = Ordinary Least Squares

GLMM = Generalized Linear Mixed Model

QReg = Quantile Regression

Quantile 1 = 0.02 corresponding to a medication possession ratio (MPR) of 40%

Quantile 2 = 0.04 corresponding to a medication possession ratio (MPR) of 60%

Quantile 3 = 0.15 corresponding to a medication possession ratio (MPR) of 80%

Quantile 4 = 0.27 corresponding to a medication possession ratio (MPR) of 90%

On the other hand, in the unadjusted model (see additional file 1, table S3), compared to NHB, NHW had 16.67% (p < 0.001) higher MPR in the first quantile (Q1), 9.47% (p < 0.001) in the second quantile (Q2), 4.76% (p < 0.001) in the third quantile and 2.63% (p < 0.001) in the fourth quantile (Q4). Similarly, compared to Other race groups, NHW had 16.67% (p < 0.001) higher MPR in the first quantile (Q1) and 8.087% (p = 0.004) in the second quantile (Q2). The mean MPR values were also higher for NHW compared to NHB (1.99%, p < 0.001) as shown in the results for OLS and GLMM. However, the mean MPR difference between NHW and Other races was not significant (0.103%, p = 0.769). Age showed a statistically significant quadratic relationship with MPR across all quantiles in the QReg as well as in the OLS and GLMM models. Divorced veterans had statistically significantly lower MPRs in quantiles 2, 3 and 4 while single veterans had lower MPRs in quantiles 2 and 4. Veterans who were employed had higher MPR compared to unemployed veterans (quantiles 1 and 3), while retired veterans had higher MPRs in quantiles 3 and 4 compared their unemployed counterparts. Veterans with a diagnosis of cancer had lower MPRs in the first two quantiles while veterans diagnosed with CHD had higher MPRs in these two quantiles and veterans with hypertension had lower MPRs in the highest quantile only compared to their counterparts without these comorbidities. Poor HbA1c control was positively associated with MPR in the first two quantiles (i.e., veterans in poor control had higher MPR in quantiles 1 and 2) but negatively associated with MPR in quantile 4 (i.e., veterans with poor control had lower MPR). Substance use disorder showed a statistically significant relationship with MPR in the lowest and the two highest quantiles but not in the second. In contrast, both OLS and GLMM did not show significant differences by gender, cancer or CHD, missing the significant differences in the lower tail of the distribution of MPR (Q1 or Q2). Table 3 shows the adjusted model from the bootstrap studies. The interpretation of the regression coefficients is similar to those in Table 2 except that these are values averaged over 10,000 bootstrapped datasets. These are computed to address concerns with regard to possible underestimation of the asymptotic standard errors (ASE) from QReg and to facilitate comparison among the different approaches. As expected, the bootstrap standard errors were larger than the ASEs but the conclusions were qualitatively similar (see Table 1). Across all quantiles except the lowest quantile (Q1), NHB had statistically significantly lower MPR in the 3^rd ^and 4^th ^quantiles compared to NHW holding all other variables constant. For example, in Q2 NHB had lower MPR compared to NHW with a difference of -4.5% (95% CI:-10.9%,1.7%). Similarly, the differences were 3.0% (-2.9%,-5.6%) and -1.9% (-3.3%,-0.54%) in Q3 and Q4, respectively.

**Table 3:** Mean parameter estimates (β) with corresponding 2.5% and 97.5% quantiles from a bootstrap study of 10,000 replications with sample size n = 5000

	*QReg*					
	***Quantile1***	***Quantile2***	***Quantile3***	***Quantile4***	***OLS***	***GLMM***

Parameter	***β (95%CI)***	***β (95%CI)***	***β (95%CI)***	***β (95%CI)***	***β (95%CI)***	***β (95%CI)***

Intercept	39.83 (14.7,61.3)	48.8(28.4,69.2)	71.5(60.5,81.5)	82.5(76.4,88.8)	88.23(84.4,91.9)	92.21(88.7,95.2)

NHB	-3.54(-11.2,3.43)	-4.55(-10.9,1.7)	-2.91(-5.6,-0.19)	-1.87(-3.3,-0.54)	-1.38(-2.31,-0.46)	-1.42(-2.10,-0.77)

Other	-5.94(-14.4,2.2)	-4.38(-13.2,3.1)	-1.05(-3.8,1.60)	0.44(-1.2,2.1)	-0.12(-1.10,0.85)	-0.65(-1.49,0.11)

NHW (REF)						

Male	-11.8(-22.8,0.39)	-6.78(-16.6,3.6)	0.61(-4.9,7.8)	1.13(-3.2,5.5)	-0.51(-2.88,2.01)	-0.55(-2.50,1.50)

Age	0.24(-0.01,0.55)	0.27(0.01,0.52)	0.17(0.05,0.29)	0.13(0.06,0.19)	0.08(0.03,0.12)	0.04(0.01,0.08)

Age^2^	-0.01(-0.02,0.01)	-0.01(-0.02,0.01)	-0.01(-0.02,0.002)	-0.01(-0.01,-0.001)	-0.01(-0.01,0.00)	-0.01(-0.01,-0.001)

Never Married	-3.51(-12.9,6.9)	-5.69(-15.9,6.6)	-0.76(-5.3,2.9)	-2.09(-5.1,0.91)	-0.88(-2.71,0.88)	-0.83(-0.41,2.12)

Divorced	-3.45(-9.7,2.2)	-4.67(-10.9,1.2)	-3.13(-5.9,-0.57)	-2.57(-4.0,-1.1)	-1.44(-2.36,-0.56)	-0.91(-0.24,1.59)

Married (REF)						

Employed	5.10(-1,8,13.1)	4.58(-2.8,11.4)	2.60(-0.17,5.42)	1.16(-4.57,2.9)	1.27(0.20,2.33)	0.08(-0.85,0.97)

Retired	-0.06(-6.9,7.8)	1.42(-5.2,7.5)	1.68(-0.91,4.36)	1.22(-0.15,2.6)	0.63(-0.27,1.53)	-0.65(-1.35,0.00)

Unemployed (REF)						

Cancer	-8.20(-19.2,2.7)	-11.4(-23.5,1.7)	-3.14(-9.9,2.99)	-0.45(-3.6,2.2)	-1.37(-3.45,0.57)	-0.45(-1.89,0.81)

CHD	11.8(0.82,21.2)	8.27(0.94,15.6)	0.65(-2.6,4.32)	0.20(-1.9,2.2)	0.70(-0.64,2.04)	-0.14(-1.04,0.79)

CHF	0.68(-10.4,11.2)	-0.29(-7.8,7.4)	-1.05(-4.7,2.46)	-2.05(-4.7,0.28)	-0.87(-2.45,0.66)	-0.79(-1.93,0.27)

Hypertension (ICD)	1.36(-6.7,11.2)	-0.89(-8.6,6.8)	-1.30(-4.7,1.54)	-1.69(-3.5,0.05)	-1.13(-2.33,0.05)	-0.09(-0.88,0.67)

Poor HbA1c	10.2(0.5,18.2)	6.45(0.34,12.0)	-0.84(-3.6,1.72)	-2.59(-4.3,-0.99)	-1.00(-2.03,0.02)	-4.13(-4.89,-3.34)

Stroke	-0.76(-12.3,10.9)	-4.04(-13.3,5.9)	-1.18(-10.3,4.29)	-0.48(-4.2,2.6)	-0.60(-2.87,1.55)	-0.05(-1.47,1.27)

Bipolar Disorder	-3.68(-24.3,19.9)	-1.57(-22.7,18.3)	1.49(-4.5,7.1)	-0.35(-4.9,3.9)	-0.24(-3.35,2.50)	-0.31(-1.71,2.06)

GAD	3.89 (-9.5,18.9)	0.50(-14.7,13.2)	2.36(-5.9,7.9)	1.57(-1.5,3.9)	0.88(-1.58,3.12)	0.74(-0.86,2.36)

Psychoses	5.90(-12.0,22.4)	4.72(-12.7, 17.1)	-2.38(-8.9,4.6)	-0.07(-6.1,3.7)	0.001(-2.7,2.6)	-0.65(-2.78,1.23)

PTSD	0.13(-11.7,12.5)	-0.75(-13.2, 9.5)	2.30(-3.1,6.5)	1.50(-0.72,3.5)	0.68(-1.01,2.20)	0.58(-0.66,1.86)

Substance Use	5.65(-3.1,15.2)	5.53(-2.5,12.7)	2.90(-0.001,5.7)	1.41(-0.06,2.8)	1.00(-0.01,2.00)	0.26(-0.48,0.99)

NHB = Non-Hispanic Black (Reference = Non-Hispanic White, NHW)

Other = Other race groups

β = Mean bootstrapped Parameter Estimate

Quantile 1 = 0.02 corresponding to a medication possession ratio (MPR) of 40%

Quantile 2 = 0.04 corresponding to a medication possession ratio (MPR) of 60.%

Quantile 3 = 0.15 corresponding to a medication possession ratio (MPR) of 80.%

Quantile 4 = 0.27 corresponding to a medication possession ratio (MPR) of 90%

95% CI = 95% confidence interval based on 2.5% and 97.5% quantiles from a bootstrap study

**Figure 3:**
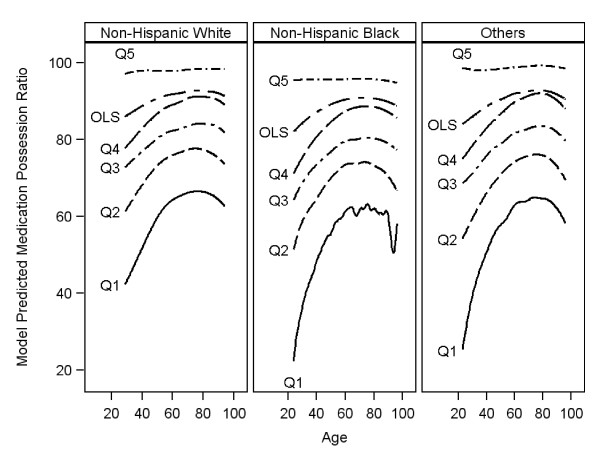
**Distribution of Predicted Mean Medication Possession Ratio (MPR) by age for each type of model (Quantile Regression versus OLS)**. OLS = ordinary least squares. Q_i _= ith quantile (i = 1,.5): Quantiles are based on unconditional MPR cutoff values: 0.33, 0.48, 0.61 0.72 and 0.94.

An additional set of analyses were performed using the second set of quantiles determined from the distribution of MPR or Scenario 2 (see Figure 3 and additional file 1, additional tables S1, S3a, and S4a). Overall, the results were qualitatively similar. Additional tables with bootstrapped based parameter estimates and corresponding 95% CI are reported (see additional file 1, tables S2, S3b, and S4b).

## Discussion

The findings of this study show that the choice of regression methods in the study of non-normal, semi-continuous and bounded responses can influence whether disparities between different racial groups are uncovered. In this large cohort of Veterans with diabetes, differences in the lower tails of the distribution of MPR by race and comorbidities such as CHD may not have been discovered using OLS or GLMM methods, but were identified using quantile regression. While the regression coefficients of race in both, OLS and GLMM, only indicate the differences in mean MPR (i.e. covariate effect in the central portion of the MPR distribution), the most clinically relevant differences that were found in the tails of the distribution of MPR (those that are low or high in adherence) were only detected through testing of the significance of the regression coefficients in the lower and upper quantiles of the QReg model.

This study used a large cohort of veterans and appropriate statistical methodology permitting a more comprehensive assessment of differences in medication non-adherence by race/ethnicity. Ordinary least squares regression, logistic regression (after categorization) and general linear mixed models assume that covariates affect only the location of the conditional distribution of the response, and not its scale or any other aspect of its distributional shape, while quantile regression has the flexibility for modeling of data with heterogeneous conditional distributions. QReg provides a complete picture of the covariate effect when a set of percentiles is modeled, and thus offers the capability to capture important features of the data possibly missed by models that average over the conditional distribution. One other recent approach that might be able to capture the effect of covariates on the entire density of MPR is Bayesian density regression (BDR) [32,33]. Like QReg, BDR avoids the assumption of normality and linearity. However, this approach is not as easy to understand and implement as QReg. Other approaches include Quasi-likelihood [32], Box-Cox transformation to normality [33] and robust regression [34,35]. However, each of these methods has its own limitations [30].

Research on medication adherence patterns has consistently shown greater non-adherence to anti-hyperglycemic agents among NHB with type 2 diabetes compared to NHW [5,9,12-14]. Consistent with prior studies, this study found that NHB were more likely to be medication non-adherent across each of the quantiles. Potential reasons for the difference in medication adherence by race/ethnicity group have been studied and seem to suggest that Blacks express more concern about drug side effects [36], medication dependency, reduced quality of life [37], and issues related to cost of medications [7,36,38-40]. For example, among an insured cohort with pharmacy benefits, an increased patient cost share of $5/month led to a 15% decrease in the odds of medication adherence and worsened glycemic control [38]. However, in the VA system where cost of medications is less of an issue because copays are very low, other factors beyond cost of medications are likely to explain the observed differences. Potential explanatory factors that were not available in our dataset include patient-level factors such as health literacy, numeracy, self-efficacy, cultural beliefs and attitudes about medications, and social support. The contribution of these and other factors need to be explored in future studies.

Despite the strengths of our data and methodology, there were limitations that need mentioning. The dataset did not include information to determine the duration of diabetes as a way to distinguish between new and regular users of diabetes medication, thus, we were not able to assess its impact on medication adherence rates. However, we created a 'new users' group who did not use medication within the first year of the study and their proportions were not different from the overall sample proportion either by race or other demographic factors (see additional file 1, tables S5). Due to the age and gender distribution of our sample, our results should be interpreted with caution in women and younger aged individuals. In addition, our findings could have been biased by the 8.6% of veterans with missing race data. While we believe that the unreported race information is missing at random, we also performed a sensitivity analysis via multiple imputation and found that the results were not different from what is reported in this paper. While the conclusions are mainly applicable to skewed and bounded outcomes from cross sectional studies, the message is easily transferable to the analysis of longitudinal skewed and bounded outcomes via longitudinal quantile regression.

## Conclusions

In conclusion, quantile regression allowed modeling the differential patterns in medication adherence between the racial/ethnic groups that would have been missed using traditional regression methods. QReg is a very useful tool for data that are heterogeneous in the sense that the tails and the central location of the conditional distributions vary differently with the covariates. Indeed, without performing quantile regression at different quantiles, an investigator would be unable to assess whether there might be a difference in these relationships. This method is also robust as it makes no distributional assumption about the error term in the model. Future studies need to be cautious when using traditional regression methods in modeling quasi-continuous and bounded outcome such as MPR.

## Abbreviations

HbA1c: Hemoglobin A1c; VA: Veterans Administration; CVD: Cardiovascular disease; CHD: Coronary heart Disease; CHF: Congestive Heart Failure; MDD: Major Depressive Disorder; ICD-9: International Classification of Diseases, Ninth Revision; VHA: Veterans Health Administration; DSS: Decision Support System; SSN: Social Security Number; DRG: Diagnostic Related Group; IRB: Institutional review board; CI: Confidence Interval; VADT: Veterans Affairs Diabetes Trial; MPR: Medication Possession Ratio; GAP: Gap between refills; CMA: Continuous Measure Of Medication Acquisition; CMOS: Continuous Multiple Interval Of Oversupply; MRA: Medication Refill Adherence; NIDDK: National Institute of Diabetes and Digestive and Kidney Diseases; OLS: Ordinary Least Squares; GLMM: General Linear Mixed Model; QReg: Quantile Regression; NHB: Non Hispanic Black; NHW: Non Hispanic White

## Conflict of interests

The authors declare that they have no competing interests.

## Authors' contributions

All authors read and approved the final manuscript. Study concept and design: LEE, MG; acquisition of data: LEE; analysis and interpretation of data: LEE, MG, MM, GG, CE, and YZ; drafting of the manuscript: MG, CPL, and MM; critical revision of the manuscript for important intellectual content: LEE, MG, CPL; study supervision: LEE.

## Pre-publication history

The pre-publication history for this paper can be accessed here:

http://www.biomedcentral.com/1471-2288/11/88/prepub

## Supplementary Material

Additional file 1 **Table S1 Adjusted parameter estimates (β) and p-values for quantile regression, ordinary least-squares regression, and the generalized linear mixed model (scenario 2)**. Table S2. Adjusted parameter estimates (β) and bootstrapped 95% CI for quantile regression, ordinary least-squares regression, and generalized linear mixed model with corresponding 2.5% and 97.5% quantiles from a bootstrap study of 10,000 replications with sample size n = 5000. Table S3ab. S3a Title: Unadjusted parameter estimates (β) and p-values for quantile regression (QReg), ordinary least-squares regression (OLS), and generalized linear mixed model (GLMM) for the MPR data with sample size n = 11,272.. S3b Title: Unadjusted parameter estimates (β), and bootstrapped 95% CI for quantile regression (QReg), ordinary least-squares regression, and generalized linear mixed model with corresponding 2.5% and 97.5% quantiles from a bootstrap study of 10,000 replications with sample size n = 5000. Table S4ab. S4a Title: Unadjusted parameter estimates (β) and p-values for quantile regression (QReg), ordinary least-squares regression (OLS), and generalized linear mixed model (GLMM) for the MPR data with sample size n = 11,272.. S4b Title: Unadjusted parameter estimates (β), and bootstrapped 95% CI for quantile regression (QReg), ordinary least-squares regression, and generalized linear mixed model with corresponding 2.5% and 97.5% quantiles from a bootstrap study of 10,000 replications with sample size n = 5000. Table S5. Comparison of the proportion of new medication users by demographic variables with the overall proportion in the study sample (washout analysis) Click here for file
