# Supplementary material for: Using quantile regression to investigate racial disparities in medication non-adherence
Source: BMC Med Res Methodol. 2011 Jun 6;11:88. doi: 10.1186/1471-2288-11-88 (PMC3121729; doi:10.1186/1471-2288-11-88)
Supplement: Additional file 1 — Table S1 Adjusted parameter estimates (β) and p-values for quantile regression, ordinary least-squares regression, and the generalized linear mixed model (scenario 2). Table S2. Adjusted parameter estimates (β) and bootstrapped 95% CI for quantile regression, ordinary least-squares regression, and generalized linear mixed model with corresponding 2.5% and 97.5% quantiles from a bootstrap study of 10,000 replications with sample size n = 5000. Table S3ab. S3a Title: Unadjusted parameter estimates (β) and p-values for quantile regression (QReg), ordinary least-squares regression (OLS), and generalized linear mixed model (GLMM) for the MPR data with sample size n = 11,272.. S3b Title: Unadjusted parameter estimates (β), and bootstrapped 95% CI for quantile regression (QReg), ordinary least-squares regression, and generalized linear mixed model with corresponding 2.5% and 97.5% quantiles from a bootstrap study of 10,000 replications with sample size n = 5000. Table S4ab. S4a Title: Unadjusted parameter estimates (β) and p-values for quantile regression (QReg), ordinary least-squares regression (OLS), and generalized linear mixed model (GLMM) for the MPR data with sample size n = 11,272.. S4b Title: Unadjusted parameter estimates (β), and bootstrapped 95% CI for quantile regression (QReg), ordinary least-squares regression, and generalized linear mixed model with corresponding 2.5% and 97.5% quantiles from a bootstrap study of 10,000 replications with sample size n = 5000. Table S5. Comparison of the proportion of new medication users by demographic variables with the overall proportion in the study sample (washout analysis) [file 1471-2288-11-88-S1.DOCX]

**Additional Table S1. Adjusted parameter estimates (β) and p-values for quantile regression, ordinary least-squares regression, and the generalized linear mixed model (scenario 2)**

|  | *Quantile 1* | | *Quantile 2* | | *Quantile 3* | | *Quantile 4* | | *Quantile 5* | | *OLS* | | *GLMM* | |
| --- | --- | --- | --- | --- | --- | --- | --- | --- | --- | --- | --- | --- | --- | --- |
| *Parameter* | ***β*** | ***P*** | ***β*** | ***P*** | ***β*** | ***P*** | ***β*** | ***P*** | ***β*** | ***P*** | ***β*** | ***P*** | ***β*** | ***P*** |
| Intercept | 48.731 | 0.000 | 65.329 | 0.000 | 72.109 | 0.000 | 80.183 | 0.000 | 99.228 | 0.000 | 88.219 | 0.000 | 89.471 | 0.000 |
| NHB | -4.054 | 0.020 | -2.946 | 0.020 | -3.067 | 0.001 | -1.833 | 0.001 | -2.073 | 0.000 | -1.377 | 0.000 | -1.278 | 0.000 |
| Other | -1.695 | 0.338 | -1.537 | 0.231 | -0.924 | 0.340 | 0.264 | 0.650 | 0.265 | 0.027 | -0.121 | 0.735 | -0.127 | 0.704 |
| NHW (REF) |  |  |  |  |  |  |  |  |  |  |  |  |  |  |
| Sex | -4.630 | 0.302 | -1.257 | 0.699 | 0.422 | 0.863 | 1.763 | 0.232 | 0.012 | 0.970 | -0.501 | 0.581 | -0.416 | 0.620 |
| Age | 0.305 | 0.000 | 0.182 | 0.001 | 0.156 | 0.000 | 0.140 | 0.000 | 0.007 | 0.155 | 0.075 | 0.000 | 0.059 | 0.000 |
| Age^2^ | -0.012 | 0.007 | -0.008 | 0.013 | -0.004 | 0.105 | -0.005 | 0.001 | 0.000 | 0.127 | -0.003 | 0.001 | -0.003 | 0.000 |
| Never Married | -7.464 | 0.017 | 0.472 | 0.835 | -0.451 | 0.792 | -2.495 | 0.015 | -0.216 | 0.307 | -0.883 | 0.162 | 0.900 | 0.130 |
| Divorced | -3.043 | 0.060 | -4.259 | 0.000 | -2.851 | 0.001 | -2.614 | 0.000 | -0.551 | 0.000 | -1.444 | 0.000 | 1.226 | 0.000 |
| Married (REF) |  |  |  |  |  |  |  |  |  |  |  |  |  |  |
| Employed | 5.671 | 0.003 | 3.230 | 0.022 | 2.752 | 0.009 | 1.076 | 0.091 | 0.073 | 0.577 | 1.272 | 0.001 | 0.579 | 0.147 |
| Retired | 1.749 | 0.296 | 2.301 | 0.058 | 1.893 | 0.039 | 1.248 | 0.023 | 0.014 | 0.901 | 0.632 | 0.063 | -0.637 | 0.037 |
| Unemployed (REF) |  |  |  |  |  |  |  |  |  |  |  |  |  |  |
| Cancer | -11.357 | 0.001 | -4.988 | 0.044 | -3.067 | 0.100 | -0.785 | 0.484 | -0.394 | 0.088 | -1.379 | 0.046 | -0.982 | 0.109 |
| CHD | 6.878 | 0.009 | 3.205 | 0.095 | 0.006 | 0.997 | 0.224 | 0.797 | -0.743 | 0.000 | 0.701 | 0.191 | 0.476 | 0.311 |
| CHF | -0.401 | 0.895 | 0.045 | 0.984 | -1.025 | 0.538 | -2.461 | 0.014 | -1.055 | 0.000 | -0.858 | 0.164 | -0.934 | 0.082 |
| Hypertension | -2.410 | 0.259 | -1.877 | 0.226 | -0.776 | 0.506 | -1.659 | 0.018 | -1.885 | 0.000 | -1.136 | 0.009 | -0.902 | 0.018 |
| Poor HbA1c | 4.648 | 0.021 | 1.242 | 0.397 | -0.614 | 0.579 | -2.629 | 0.000 | -3.167 | 0.000 | -0.997 | 0.015 | -4.982 | 0.000 |
| Stroke | -5.396 | 0.199 | -5.451 | 0.073 | -0.548 | 0.811 | 0.102 | 0.941 | -0.874 | 0.002 | -0.617 | 0.469 | -0.543 | 0.466 |
| Bipolar | 2.980 | 0.594 | 3.593 | 0.375 | 2.302 | 0.451 | -0.561 | 0.760 | -0.687 | 0.070 | -0.223 | 0.844 | 0.084 | 0.932 |
| GAD | -2.157 | 0.652 | 2.602 | 0.454 | 3.664 | 0.161 | 1.675 | 0.287 | -0.102 | 0.752 | 0.883 | 0.362 | 0.631 | 0.450 |
| Psychoses | 3.269 | 0.510 | -2.509 | 0.486 | -3.385 | 0.212 | 0.082 | 0.960 | -1.571 | 0.000 | 0.002 | 0.998 | -0.546 | 0.528 |
| PTSD | 0.653 | 0.839 | 1.181 | 0.612 | 2.603 | 0.138 | 1.862 | 0.077 | 0.041 | 0.849 | 0.667 | 0.304 | 0.869 | 0.124 |
| Substance Use | 5.649 | 0.008 | 4.895 | 0.001 | 2.615 | 0.024 | 1.710 | 0.014 | -0.222 | 0.123 | 0.993 | 0.021 | 0.842 | 0.025 |

NHB = Non-Hispanic Black (Reference= Non-Hispanic White, NHW)

Other = Other race groups

β = Parameter Estimate

P = p-value

OLS = Ordinary Least Squares

GLMM = Generalized Linear Mixed Model

Quantile 1 = 0.05 corresponding to a medication possession ratio (MPR) of 66.7%

Quantile 2 = 0.10 corresponding to a medication possession ratio (MPR) of 75.0%

Quantile 3 = 0.15 corresponding to a medication possession ratio (MPR) of 80.0%

Quantile 4 = 0.25 corresponding to a medication possession ratio (MPR) of 88.9%

Quantile 5 = 0.50 corresponding to a medication possession ratio (MPR) of 97.7%

**Additional Table S2. Adjusted parameter estimates (β) and bootstrapped 95% CI for quantile regression, ordinary least-squares regression, and generalized linear mixed model with corresponding** **2.5% and 97.5% quantiles from a bootstrap study of 10,000 replications with sample size n=5000**

|  | *Quantile1* | | *Quantile2* | | *Quantile3* | | *Quantile4* | | *Quantile5* | | *OLS* | | *GLMM* | |
| --- | --- | --- | --- | --- | --- | --- | --- | --- | --- | --- | --- | --- | --- | --- |
| *Parameter* | *β* | *95%CI* | *β* | *95%CI* | *β* | *95%CI* | *β* | *95%CI* | *β* | *95%CI* | *β* | *95%CI* | *β* | *95%CI* |
| Intercept | 52.59 | (34.96,70.99) | 64.23 | (50.55,77.19) | 71.53 | (60.54,81.54) | 80.96 | (74.61,87.62) | 99.79 | (98.00,100.00) | 88.23 | (84.41, 91.99) | 92.21 | (88.72, 95.26) |
| Non-Hispanic Black | -3.82 | (-9.79,1.41) | -3.05 | (-6.58,0.40) | -2.91 | (-5.56,-0.19) | -1.91 | (-3.40,-0.44) | -1.40 | (-2.02,-0.78) | -1.38 | (-2.32, -0.46) | -1.43 | (-2.10, -0.77) |
| Other Race | -2.72 | (-9.29,1.90) | -2.08 | (-6.80,2.02) | -1.05 | (-3.75,1.60) | 0.18 | (-1.76,1.95) | 0.00 | (0.00,0.00) | -0.12 | (-1.10, 0.85) | -0.65 | (-1.49, 0.11) |
| Non-Hispanic White (REF) |  |  |  |  |  |  |  |  |  |  |  |  |  |  |
| Male | -4.32 | (-13.56,5.59) | -0.67 | (-7.86,6.89) | 0.61 | (-4.88,7.84) | 1.18 | (-3.19,5.58) | 0.21 | (0.00,2.00) | -0.51 | (-2.88, 2.01) | -0.55 | (-2.45, 1.50) |
| Age | 0.24 | (0.01,0.46) | 0.19 | (0.04,0.35) | 0.17 | (0.05,0.29) | 0.14 | (0.06,0.20) | 0.00 | (0.00,0.00) | 0.08 | (0.03, 0.12) | 0.04 | (0.01, 0.08) |
| Age (Squared) | -0.01 | (-0.02,0.01) | -0.01 | (-0.02,0.00) | -0.01 | (-0.01,0.00) | 0.00 | (-0.01,0.00) | 0.00 | (0.00,0.00) | -0.01 | (-0.006, 0.01) | -0.01 | (-0.01, -0.01) |
| Never Married | -5.17 | (-15.72,6.62) | -0.82 | (-9.04,5.18) | -0.76 | (-5.25,2.90) | -2.11 | (-4.96,1.00) | -0.05 | (-0.69,0.00) | -0.88 | (-2.71, 0.88) | 0.83 | (-0.41, 2.12) |
| Divorced | -3.84 | (-9.65,1.26) | -3.90 | (-7.13,-0.57) | -3.12 | (-5.88,-0.57) | -2.71 | (-4.28,-1.13) | -0.01 | (0.00,0.00) | -1.44 | (-2.36, -0.56) | 0.91 | (0.24, 1.59) |
| Married (REF) |  |  |  |  |  |  |  |  |  |  |  |  |  |  |
| Employed | 4.75 | (-1.03,10.22) | 3.37 | (-0.46,7.22) | 2.60 | (-0.17,5.42) | 1.17 | (-0.64,3.02) | 0.00 | (0.00,0.00) | 1.27 | (0.20, 2.34) | 0.08 | (-0.85, 0.97) |
| Retired | 1.72 | (-2.93,6.61) | 1.96 | (-1.70,5.70) | 1.68 | (-0.91,4.36) | 1.22 | (-0.28,2.70) | 0.00 | (0.00,0.00) | 0.627 | (-0.27, 1.53) | -0.65 | (-1.35, 0.00) |
| Unemployed (REF) |  |  |  |  |  |  |  |  |  |  |  |  |  |  |
| Cancer | -10.16 | (-23.83,2.56) | -4.51 | (-11.88,2.59) | -3.14 | (-9.98,2.99) | -0.58 | (-4.00,2.32) | -0.56 | (-1.64,0.00) | -1.37 | (-3.45, 0.57) | -0.45 | (-1.89, 0.81) |
| CHD | 6.71 | (0.09,13.58) | 2.29 | (-2.60,6.86) | 0.64 | (-2.58,4.32) | 0.37 | (-1.92,2.63) | -0.44 | (-1.48,0.05) | 0.70 | (-0.64, 2.04) | -0.14 | (-1.04, 0.79) |
| CHF | -0.08 | (-7.26,7.16) | 0.30 | (-5.30,5.20) | -1.05 | (-4.67,2.46) | -2.20 | (-4.97,0.46) | -1.32 | (-2.48,-0.19) | -0.87 | (-2.45, 0.66) | -0.79 | (-1.93, 0.27) |
| Hypertension (ICD defined) | -1.80 | (-8.48,4.64) | -1.78 | (-5.90,2.16) | -1.30 | (-4.65,1.54) | -1.73 | (-3.72,0.10) | -1.36 | (-2.22,-0.47) | -1.13 | (-2.33, 0.05) | -0.09 | (-0.88, 0.67) |
| Poor HbA1c Control | 4.36 | (-0.45,9.00) | 0.55 | (-2.76,3.86) | -0.84 | (-3.60,1.71) | -2.48 | (-4.25,-0.77) | -2.46 | (-3.33,-1.60) | -1.00 | (-2.03, 0.02) | -4.13 | (-4.89, -3.34) |
| Stroke | -4.25 | (-13.39,5.30) | -3.71 | (-12.63,5.78) | -1.18 | (-10.31,4.31) | -0.46 | (-4.60,2.76) | -0.68 | (-2.32,0.66) | -0.60 | (-2.87, 1.55) | -0.05 | (-1.47, 1.27) |
| Bipolar Disorder | 0.20 | (-21.09,16.93) | 3.32 | (-9.79,10.24) | 1.49 | (-4.53,7.08) | -0.24 | (-5.35,4.33) | -1.07 | (-3.29,0.09) | -0.24 | (-3.35, 2.50) | 0.31 | (-1.71, 2.06) |
| Generalized Anxiety Disorder | -0.13 | (-15.60,12.39) | 1.21 | (-10.17,9.91) | 2.36 | (-5.95,7.88) | 1.77 | (-1.78,4.38) | -0.27 | (-1.59,0.03) | 0.88 | (-1.58, 3.12) | 0.74 | (-0.86, 2.36) |
| Psychoses | 2.78 | (-9.93,14.69) | -2.00 | (-9.99,6.18) | -2.38 | (-8.98,4.60) | -0.49 | (-6.90,3.92) | -1.21 | (-3.25,0.01) | 0.01 | (-2.68, 2.56) | -0.65 | (-2.78, 1.23) |
| PTSD | -0.24 | (-11.51,8.53) | 1.07 | (-6.24,7.59) | 2.30 | (-3.07,6.49) | 1.71 | (-0.99,3.92) | -0.05 | (-0.74,0.00) | 0.68 | (-1.01, 2.20) | 0.58 | (-0.66, 1.86) |
| Substance Use Disorder | 5.32 | (-1.52,11.38) | 4.66 | (0.82,8.01) | 2.90 | (0.00,5.74) | 1.69 | (0.13,3.21) | -0.13 | (-0.85,0.00) | 1.00 | (-0.01, 2.00) | 0.26 | (-0.48, 0.99) |

Quantile 1 = 0.05 corresponding to a medication possession ratio (MPR) of 66.7%

Quantile 2 = 0.10 corresponding to a medication possession ratio (MPR) of 75.0%

Quantile 3 = 0.15 corresponding to a medication possession ratio (MPR) of 81.6%

Quantile 4 = 0.25 corresponding to a medication possession ratio (MPR) of 88.9%

Quantile 5 = 0.50 corresponding to a medication possession ratio (MPR) of 97.7%

NHB = Non-Hispanic Black (Reference= Non-Hispanic White, NHW)

Other = Other race groups

β = Parameter Estimate

OLS = Ordinary Least Squares

GLMM = Generalized Linear Mixed Model

**Additional Table S3a. Unadjusted parameter estimates (β) and p-values for quantile regression (QReg), ordinary least-squares regression (OLS), and generalized linear mixed model (GLMM) for the MPR data with sample size n=11,272.**

|  | ***QReg*** | | | | | | | | | |  | |
| --- | --- | --- | --- | --- | --- | --- | --- | --- | --- | --- | --- | --- |
| ***Parameter*** | ***Quantile 1*** | | ***Quantile 2*** | | ***Quantile 3*** | | ***Quantile 4*** | | ***OLS*** | | ***GLMM*** | |
| **Intercept** | 50.00 | <.0001 | 63.64 | <.0001 | 83.33 | <.0001 | 90.79 | <.0001 | 92.18 | <0.001 | 92.55 | <.0001 |
| **NHB** | -16.67 | <.0001 | -9.47 | 0.0005 | -4.76 | <.0001 | -2.63 | <.0001 | -1.99 | <0.001 | -1.86 | <.0001 |
| **Other** | -16.67 | <.0001 | -8.08 | 0.0036 | -1.11 | 0.1687 | 0.51 | 0.3730 | -0.10 | 0.769 | -0.17 | 0.6076 |
| **NHW (REF)** |  |  |  |  |  |  |  |  |  |  |  |  |

Quantile 1 = 0.02 corresponding to a medication possession ratio (MPR) of 40%

Quantile 2 = 0.04 corresponding to a medication possession ratio (MPR) of 60%

Quantile 3 = 0.15 corresponding to a medication possession ratio (MPR) of 80%

Quantile 4 = 0.27 corresponding to a medication possession ratio (MPR) of 90%

NHB = Non-Hispanic Black (Reference= Non-Hispanic White, NHW)

Other = Other race groups

β = Parameter Estimate

p = p-value

OLS = Ordinary Least Squares

GLMM = Generalized Linear Mixed Model

**Table S3b. Unadjusted parameter estimates (β), and bootstrapped 95% CI for quantile regression (QReg), ordinary least-squares regression, and generalized linear mixed model with corresponding 2.5% and 97.5% quantiles from a bootstrap study of 10,000 replications with sample size n=5000**

|  | ***Quantile 1*** | | ***Quantile 2*** | | ***Quantile 3*** | | ***Quantile 4*** | | ***OLS*** | | ***GLMM*** | |
| --- | --- | --- | --- | --- | --- | --- | --- | --- | --- | --- | --- | --- |
|  | ***Β*** | ***95%CI*** | ***β*** | ***95%CI*** | ***β*** | ***95%CI*** | ***β*** | ***95%CI*** | ***β*** | ***95%CI*** | ***β*** | ***95%CI*** |
| **Intercept** | 47.44 | (33.33, 54.17) | 63.55 | (57.14, 66.67) | 83.12 | (81.48, 83.33) | 90.87 | (90.00, 91.67) | 92.18 | (91.66, 92.69) | 93.77 | (93.41,94.13) |
| **NHB** | -9.79 | (-20.00, 5.56) | -8.94, | (-16.67, -0.001) | -4.36 | (-5.88, -1.85) | -2.71 | (-4.24, -1.59) | -1.98 | (-2.91, -1.09) | -1.82 | (-2.50,-1.16) |
| **Other** | -13.01 | (-20.37, -0.001) | -8.43, | (-19.20, 2.64) | -1.05 | (-4.54, 1.28) | 0.17 | (-1.87, 1.80) | -0.10 | (-1.06, 0.84) | -0.78 | (-1.59,-0.04) |
| **NHW (REF)** |  |  |  |  |  |  |  |  |  |  |  |  |

Quantile 1 = 0.02 corresponding to a medication possession ratio (MPR) of 40%

Quantile 2 = 0.04 corresponding to a medication possession ratio (MPR) of 60%

Quantile 3 = 0.15 corresponding to a medication possession ratio (MPR) of 80%

Quantile 4 = 0.27 corresponding to a medication possession ratio (MPR) of 90%

NHB = Non-Hispanic Black (Reference= Non-Hispanic White, NHW)

Other = Other race groups

β = Parameter Estimate

OLS = Ordinary Least Squares

GLMM = Generalized Linear Mixed Model

**Additional Table S4a. Unadjusted parameter estimates (β) and p-values for quantile regression (QReg), ordinary least-squares regression (OLS), and generalized linear mixed model (GLMM) for the MPR data with sample size n=11,272.**

|  | ***QReg*** | | | | | | | | | |  | |  | |
| --- | --- | --- | --- | --- | --- | --- | --- | --- | --- | --- | --- | --- | --- | --- |
|  | ***Quantile 1*** | | ***Quantile 2*** | | ***Quantile 3*** | | ***Quantile 4*** | | ***Quantile 5*** | | ***OLS*** | | ***GLMM*** | |
| ***Parameter*** | ***β*** | ***P*** | ***β*** | ***P*** | ***β*** | ***P*** | ***β*** | ***P*** | ***β*** | ***P*** | ***β*** | ***P*** | ***β*** | ***P*** |
| **Intercept** | 66.67 | <.0001 | 77.78 | <.0001 | 83.33 | <.0001 | 90.0 | <.0001 | 97.62 | <.0001 | 92.18 | 0.000 | 92.55 | <.0001 |
| **NHB** | -5.72 | 0.0001 | -5.56 | 0.0016 | -4.76 | <.0001 | -2.96 | <.0001 | -2.06 | <.0001 | -1.98 | 0.000 | -1.85 | <.0001 |
| **Other** | -4.77 | 0.0019 | -5.56 | 0.0021 | -1.11 | 0.1687 | -0.56 | 0.3946 | 2.38 | <.0001 | -0.10 | 0.769 | -0.169 | 0.606 |
| **NHW (REF)** |  |  |  |  |  |  |  |  |  |  |  |  |  |  |

Quantile 1 = 0.05 corresponding to a medication possession ratio (MPR) of 66.7%

Quantile 2 = 0.10 corresponding to a medication possession ratio (MPR) of 75.0%

Quantile 3 = 0.15 corresponding to a medication possession ratio (MPR) of 81.6%

Quantile 4 = 0.25 corresponding to a medication possession ratio (MPR) of 88.9%

Quantile 5 = 0.50 corresponding to a medication possession ratio (MPR) of 97.7%

NHB = Non-Hispanic Black (Reference= Non-Hispanic White)

Other = Other race groups

β = Parameter Estimate

SE(β) = Standard Error

p = p-value

OLS = Ordinary Least Squares

GLMM = Generalized Linear Mixed Model

**Table S4b. Unadjusted parameter estimates (β), and bootstrapped 95% CI for quantile regression (QReg), ordinary least-squares regression, and generalized linear mixed model with corresponding 2.5% and 97.5% quantiles from a bootstrap study of 10,000 replications with sample size n=5000**

|  | ***Quantile 1*** | | ***Quantile 2*** | | ***Quantile 3*** | | ***Quantile 4*** | | ***Quantile 5*** | | ***OLS*** | | ***GLMM*** | |
| --- | --- | --- | --- | --- | --- | --- | --- | --- | --- | --- | --- | --- | --- | --- |
|  | ***β*** | ***95%CI*** | ***β*** | ***95%CI*** | ***β*** | ***95%CI*** | ***β*** | ***95%CI*** | ***β*** | ***95%CI*** | ***β*** | ***95%CI*** | ***β*** | ***95%CI*** |
| **Intercept** | 66.62 | (66.67, 66.67) | 77.02 | (75.00, 77.78) | 83.12 | (81.48, 83.33) | 89.906 | (88.89, 90.68) | 97.65 | (97.22, 98.04) | 92.18 | (91.66, 92.69) | 93.77 | (93.41,94.13) |
| **NHB** | -6.30 | (-12.50, 0.00) | -5.05 | (-10.27, -1.19) | -4.36 | (-5.88, -1.85) | -2.804 | (-4.37, -1.09) | -2.11 | (-2.75, -1.38) | -1.98 | (-2.91, -1.09) | -1.82 | (-2.50,-1.16.) |
| **Other** | -4.27 | (-11.11, 0.00) | -4.92 | (-11.11, 1.36) | -1.05 | (-4.54, 1.28) | -0.300 | (-2.05, 1.81) | 2.35 | (1.96, 2.78) | -0.10 | (-1.06, 0.84) | -0.78 | (-1.59,-0.04) |
| **NHW (REF)** |  |  |  |  |  |  |  |  |  |  |  |  |  |  |

Quantile 1 = 0.05 corresponding to a medication possession ratio (MPR) of 66.7%

Quantile 2 = 0.10 corresponding to a medication possession ratio (MPR) of 75.0%

Quantile 3 = 0.15 corresponding to a medication possession ratio (MPR) of 81.6%

Quantile 4 = 0.25 corresponding to a medication possession ratio (MPR) of 88.9%

Quantile 5 = 0.50 corresponding to a medication possession ratio (MPR) of 97.7%

NHB = Non-Hispanic Black (Reference= Non-Hispanic White)

Other = Other race groups

β = Parameter Estimate

SE(β) = Standard Error

p = p-value

OLS = Ordinary Least Squares

GLMM = Generalized Linear Mixed Model

**Additional Table S5. Comparison of the proportion of new medication users by demographic variables with the overall proportion in the study sample (washout analysis)**

| *Value* | *New Users (%)* | *Total Users (%)* |
| --- | --- | --- |
| NHW | 46.8 | 47.1 |
| NHB | 22.7 | 27.2 |
| Other/Missing Race | 30.5 | 25.8 |
| Female | 2.9 | 2.7 |
| Male | 97.1 | 97.3 |
| Never Married | 6.3 | 6.2 |
| Divorced | 66.1 | 65.2 |
| Married | 27.7 | 28.6 |
| Employed | 23.3 | 20.8 |
| Retired | 45.1 | 48.3 |
| Unemployed | 31.6 | 30.9 |
| Cancer | 4.1 | 5.0 |
| CHD | 10.8 | 14.0 |
| CHF | 5.6 | 8.1 |
| Hypertension | 20.9 | 25.8 |
| Poor HbA1c Control | 12.1 | 14.3 |
| Stroke | 2.5 | 3.0 |
| Bipolar Disorder | 1.5 | 1.9 |
| Generalized Anxiety Disorder | 1.8 | 2.2 |
| Major Depressive Disorder | 6.2 | 7.8 |
| Post-traumatic Stress Disorder | 4.6 | 5.1 |
| Psychotic Disorder | 1.9 | 2.4 |
| Substance Abuse Disorder | 11.7 | 14.4 |

NHW = Non-Hispanic White

NHB = Non-Hispanic Black

Other = Other race groups

CHD = coronary heart disease

CHF = congestive heart failure

HbA1c = glycosolated hemoglobin
